# Case Report: Preventing X-linked retinoschisis transmission via MARSALA-based PGT-M

**DOI:** 10.3389/fopht.2025.1734044

**Published:** 2026-01-14

**Authors:** Jieliang Li, Xiaojun Wen, Zhanhui Ou, Xiaowu Fang, Jing Du, Xiufeng Lin, Wanna Ke, Jiaqi Wu, Nengqing Liu

**Affiliations:** 1 Reproductive Center, Zhongshan Boai Hospital, Zhongshan, Guangdong, China; 2 The Second Clinical College, Southern Medical University, Guangzhou, Guangdong, China

**Keywords:** marsala, next-generation sequencing, preimplantation genetic testing, RS1, X-linked retinoschisis

## Abstract

X-linked retinoschisis (XLRS) is an X-linked recessive inherited retinal disease caused by mutations in the *RS1* gene. This case report describes the successful application of preimplantation genetic testing for monogenic diseases (PGT-M) to prevent the intergenerational transmission of a pathogenic *RS1* variant within a family. Prior to PGT-M, the family had a male child with XLRS who experienced bilateral visual impairment and metamorphopsia at 4 years of age. we employed next-generation sequencing (NGS) to identify a single-nucleotide variant, c.187T>C (p. Cys63Arg; NM_000330.4), in *RS1*. Subsequently, we identified the mutation in the proband and his parents, which was confirmed by Sanger sequencing. In the PGT-M cycle, eight blastocysts were biopsied and analyzed using the “Mutated allele revealed by sequencing with aneuploidy and linkage analyses (MARSALA)” platform. This involved whole-genome amplification via multiple annealing and looping-based amplification cycles (MALBAC), followed by next-generation sequencing for concurrent single-nucleotide polymorphism (SNP) haplotype analysis to track the mutant allele and comprehensive chromosomal copy number variation (CNV) screening. The analysis identified three euploid embryos (E1, E4 and E5) without the familial *RS1* mutation. A high-quality embryo (E1, 6AA) was transferred following genetic counseling, resulting in a clinical pregnancy. Mid-trimester amniocentesis confirmed a normal male karyotype and the absence of the pathogenic *RS1* variant, leading to the birth of a healthy baby. This case demonstrates that the integrated MARSALA-based PGT-M strategy is a powerful tool for families with X-linked disorders to conceive healthy offspring.

## 1 Introduction

X-linked retinoschisis (XLRS), an X-chromosome recessive genetic fundus disease with an estimated incidence of 1/5,000 to 1/25,000, is one of the main causes of macular degeneration in adolescent males (1). The causative gene of XLRS, *RS1*, is located on Xp22.1 (2) and contains six exons and five introns. *RS1* encodes a retinoschisin protein that contains a signal sequence (exons 1 and 2), an RS domain (exon 3), and a discoidin domain (exons 4–6) (3). The *RS1* protein plays a crucial role in the structural integrity of the retina as a cell adhesion protein between the photoreceptors and bipolar cells (4). Currently, more than 330 pathogenic mutations have been reported in the Human Gene Mutation Database (HGMD: https://www.hgmd.cf.ac.uk/ac/index.php), most of which are missense mutations. Pathogenic *RS1* mutations are hemizygous; retinoschisis usually occurs only in male carriers (5), and female carriers with heterozygous mutations are phenotypically normal. The clinical progression of XLRS is slow and shows great heterogeneity in the genotypes and phenotypes. Most patients are of school-going age when they develop spoke-wheel pattern foveal schisis and progressive visual impairment, which may be accompanied by manifestations such as vascular abnormalities, vitreous veils, pigment changes, inner retinal breaks, macular atrophy, and retinal detachment (6). Standard and effective treatment procedures for XLRS are lacking. Carbonic anhydrase inhibitors (CAI) are considered effective against retinitis pigmentosa (RP) and cystoid macular edema (CME) in some patients with XLRS (7) but have no significant effect on patients with advanced XLRS. Adeno-associated virus (AAV) gene therapy, By introducing, altering, or repairing specific genes, which can potentially improve the retinal phenotype in patients with XLRS, is still under investigation (8).The most effective strategy to avoid XLRS in patients is to prevent mutant *RS1* from being passed down through generations. Although genetic counseling and amniocentesis-based prenatal diagnosis have traditionally been used to prevent birth defects, preimplantation genetic testing for monogenic disorders (PGT-M) is another option for carriers of these disorders. PGT-M is a novel prenatal diagnostic method that combines assisted reproductive technology (ART) and genetic diagnosis (9).

In this case, we employed an advanced PGT-M strategy utilizing the “Mutant Allele Revealed by Sequencing Analysis (MARSALA)” platform (10). The core of this approach lies in the initial whole-genome amplification of the biopsied embryonic cell(s) using multiple annealing and looping-based amplification cycles (MALBAC). This technology provides uniform amplification of the minute quantity of DNA, which is crucial for reliable subsequent analysis. The amplified DNA was then subjected to next-generation sequencing (NGS), enabling the MARSALA workflow to concurrently perform single-nucleotide polymorphism (SNP) haplotype analysis for precise mutation tracking and comprehensive chromosomal screening. We report the case of a couple in which a woman was an asymptomatic carrier of the *RS1* variant and had a son with XRLS. We assisted the couple in conceiving a healthy baby and prevented the *RS1* variant from being passed down using this integrated MALBAC-NGS and SNP haplotype analysis approach.

## 2 Methods

### 2.1 Subjects and ethics approval

The couple had a son in 2013. When he was 4 years old, he experienced vision loss and grayish-white retinal bulges in the fundus. Ultrasonography revealed vitreous opacity and retinal detachment in both eyes. The boy was clinically suspected of having a genetic eye disease and underwent next-generation sequencing, which revealed a hemizygous variant c.187T>C (p.Cys63Arg; NM_000330.3) in *RS1*. The couple, a 31-year-old female and a 35-year-old male, desired another child and sought genetic and fertility guidance at the Reproductive Center of Zhongshan Bo’ai Hospital. They underwent clinical assessments, sample collection, preimplantation genetic testing (PGT), and other forms of ART. The internal ethics committee of Zhongshan Bo’ai Hospital approved this study, and the couple provided informed consent.

### 2.2 Genomic DNA extraction and variant site detection

Genomic DNA (gDNA) was isolated from the peripheral blood of the couple and their children using a TIANamp Blood DNA kit (Tiangen Biotechnology, Cat# DP348-02). Polymerase chain reaction (PCR) amplification and Sanger sequencing were performed to validate *RS1* mutation sites. The forward and reverse primers (synthesized by Guangzhou IGE Biotechnology) were designed using Primer 5.0 software, F:5′-TCACCTGGTGCTTGTTGAGTATTG-3′ and R:5′-GGGCCTTGTTTGCAGTCCAC-3′, to cover the coding exon and flanking introns of *RS1* for PCR amplification of the mutation. Sanger sequencing data were analyzed using ChromasPro software (Yikon Genomics). After a database and literature survey of the mutation sites, the 3D protein structure of *RS1* was assembled using the PyMOL software.

### 2.3 ART procedure and embryo biopsy

The standard long protocol for gonadotropin-releasing hormone (GnRH) agonist administration was used to stimulate ovarian activity. Subsequently, gonadotropins were injected daily from day three of the next cycle to foster adequate follicle growth. When the follicles reached 18 mm in diameter, ovulation was induced by human chorionic (hCG) injection, and the oocytes were retrieved after 36 h using ultrasound-guided follicular puncture. Following the program standards, embryos were generated using intracytoplasmic sperm injection (ICSI) and cultured until the blastocyst stage. Blastocyst formation and morphology were observed on D5 and D6 and scored using the Gardner embryo grading system. Only blastocysts with a Gardner grade of 3BB or higher underwent biopsy. Between four and ten trophectoderm (TE) cells were biopsied from each blastocyst on day 5 or day 6 by zona drilling with a laser, and transferred into a lysis buffer for whole-genome amplification (WGA).

### 2.4 Whole genome amplification

Whole genome amplification (WGA) of TE cells was performed using the MALBAC WGA kit (Yikon Genomics, XK-028-24, Suzhou, China). TE cells were transferred to a 0.2 µL PCR tube containing 4.5 µL lysis buffer (Yikon Genomics). MALBAC pre-amplification and exponential amplification were performed using the manufacturer’s instructions. We obtained 2–5 μg of DNA from each embryo biopsy sample. Product concentration was established as the quality control criterion for WGA, with the concentration measured via the Qubit 4.0 Fluorometer and samples designated as qualified if the concentration was 10ng/ul or higher.

### 2.5 The MARSALA method for SNP haplotyping and CNV analysis of each embryo

A total of 20 ng of WGA product was used for library construction by the enzyme cutting method. The 200–500 bp library was sequenced using a MGI 2000 sequencer. The average depth of sequencing was 5X. Sequencing data were analyzed for CNVs and SNP haplotyping in accordance with our in-house procedures.The original reads were aligned and compared to the UCSC hg19 Human reference genome, to filter out the low-quality and duplicate sequences for CNV analysis. All reads were counted with a 1 Mb window unit (bin) and standardized by GC content and reference data set. The number of reads increased by 50% when the number of copies per bin was increased from two to three, and decreased by 50% when the number of copies per bin was reduced from two to one. The embryos with CNVs of ≥4 Mb were reported by the circular binary segmentation (CBS) algorithm. Finally, the R language program was applied to visualize the CNVs of each bin of 24 chromosomes. The sequencing data were analyzed by Yikon Genomics Company. Prior to the analysis of the embryos, high-throughput sequencing was performed on genomic DNA from the couple and the proband to identify informative SNP markers. We specifically selected SNP loci that were heterozygous in the mother and hemizygous in the proband to establish the haplotypic linkage with the pathogenic *RS1* mutation. Using the SNP information from the whole genome of each embryo, 30 SNP markers that were located within 2 Mb upstream or downstream of the gene were selected for *RS1* SNP haplotyping. Only the SNPs that were heterozygous in one parent and homozygous in the other were considered to be informative SNPs.

## 3 Results

### 3.1 Patients and relatives

**Figure 1:**
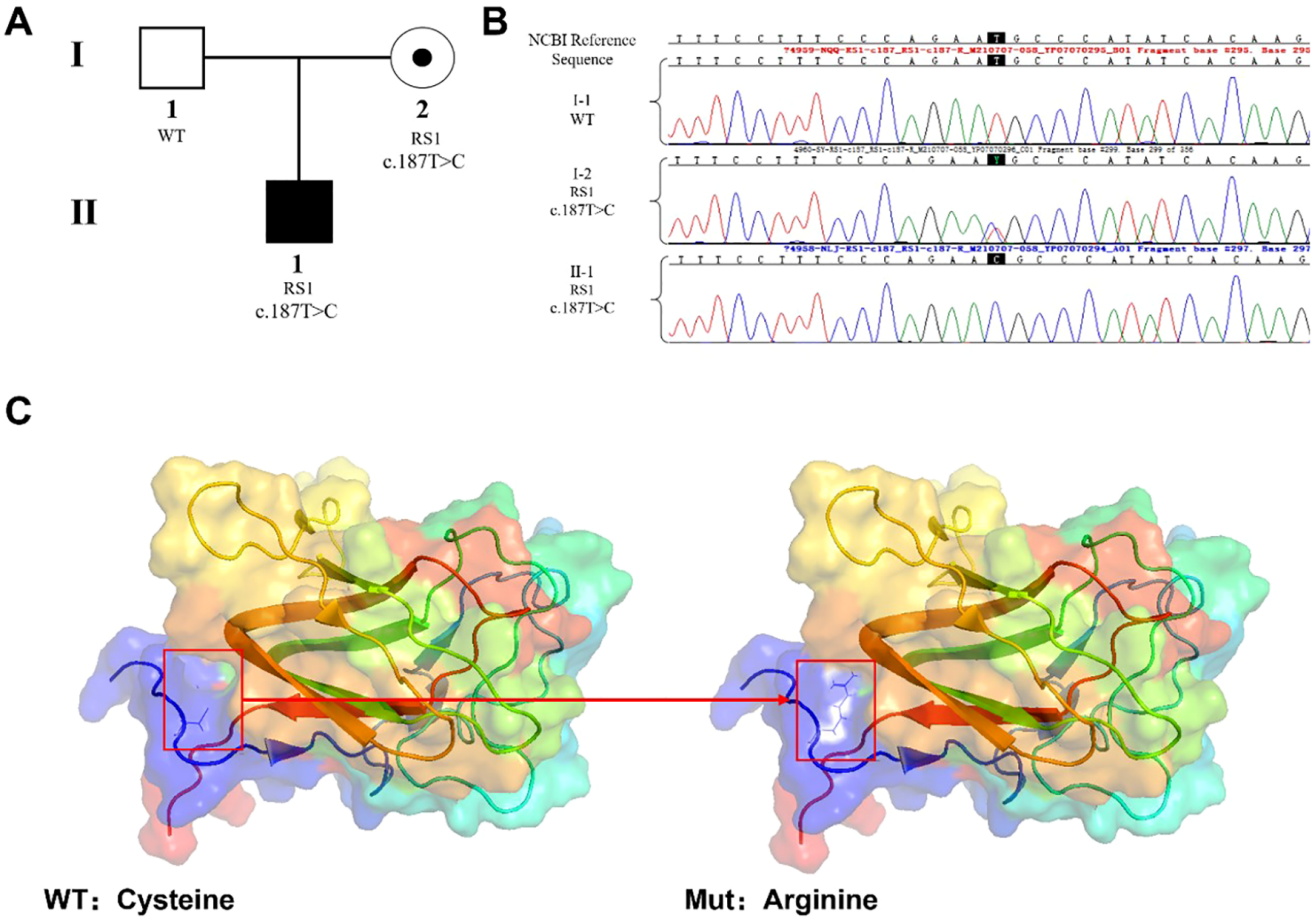
*RS1* mutation and identification of affected family members. **(A)** Pedigree of an XLRS-affected family: the proband (II-1), a 3 years old boy, has XLRS caused by a pathogenic *RS1* mutation; the mother (I-2) has the same mutation but without clinical manifestations; and the father is normal. **(B)** Sanger sequencing confirms the hemizygous *RS1* mutation c.187T>C (p. Cys63Arg; NM_000330.3) in the proband (II-1), and his mother (I-2); the father does not have any *RS1* mutations. **(C)** Crystal structure of the *RS1* protein subunit. The cysteine at position 63 in the primary structure of the protein Cysteine (WT) is replaced by Arginine (Mut) when the c.187T>C mutation occurs.

Clinical records of the proband from the referring hospital documented markedly diminished visual acuity in both eyes. Although original images were unavailable, the clinical report described characteristic findings of X-linked retinoschisis. Specifically, ultrasonography revealed vitreous opacification and putative retinal detachment in both eyes, and fundus examination documented a typical “spoke-wheel” pattern of foveal schisis in the macula. Optical coherence tomography (OCT) reports confirmed the splitting of the neurosensory retina, primarily occurring at the level of the inner nuclear and outer plexiform layers, consistent with the diagnosis of XLRS. Neither parent exhibited vision loss or organic pathological ocular changes. Peripheral blood samples were obtained from the proband and the parents for pedigree analysis. NGS results indicated that the c.187T>C mutation was present in RS1 in the proband and conformed the specifical RS1 variant in his mother by Sanger sequencing but not in his father (**Figure 1A, B**). This sequence change replaces cysteine with arginine at codon 63 of the RS1 protein (p.Cys63Arg; NM_000330.3) and likely to be disruptive (**Figure 1C**). The pathogenicity of the mutations was analyzed according to the American College of Medical Genetics and Genomics (ACMG/AMP) standards and guidelines (11). The c.187T>C mutation in *RS1* was absent from the population database (gnomAD) (PM2_Supporting). Clingen recomends the use of the REVEL score for missense variants classification, the c.187C>T variant has a REVEL score of 0.953(≥ 0.932), which provides strong evidence of pathogenicity (PP3_Strong) (12). Furthermore, the clinical phenotype of the proband was highly specific to *RS1*-related X-linked retinoschisis (PP4). Although the variant has been listed in ClinVar (ID: 1517929), implying previous observation, we prioritized the strict application of evidence criteria. Based on the ClinGen Bayesian classification framework (13), the combination of one strong piece of evidence (PP3_Strong) and two supporting pieces of evidence (PM2_Supporting and PP4) yields a total score of 6, classifying the variant as “Likely Pathogenic”.

### 3.2 Blastocyst culture, trophoblast biopsy, and Sanger sequencing

**Figure 2:**
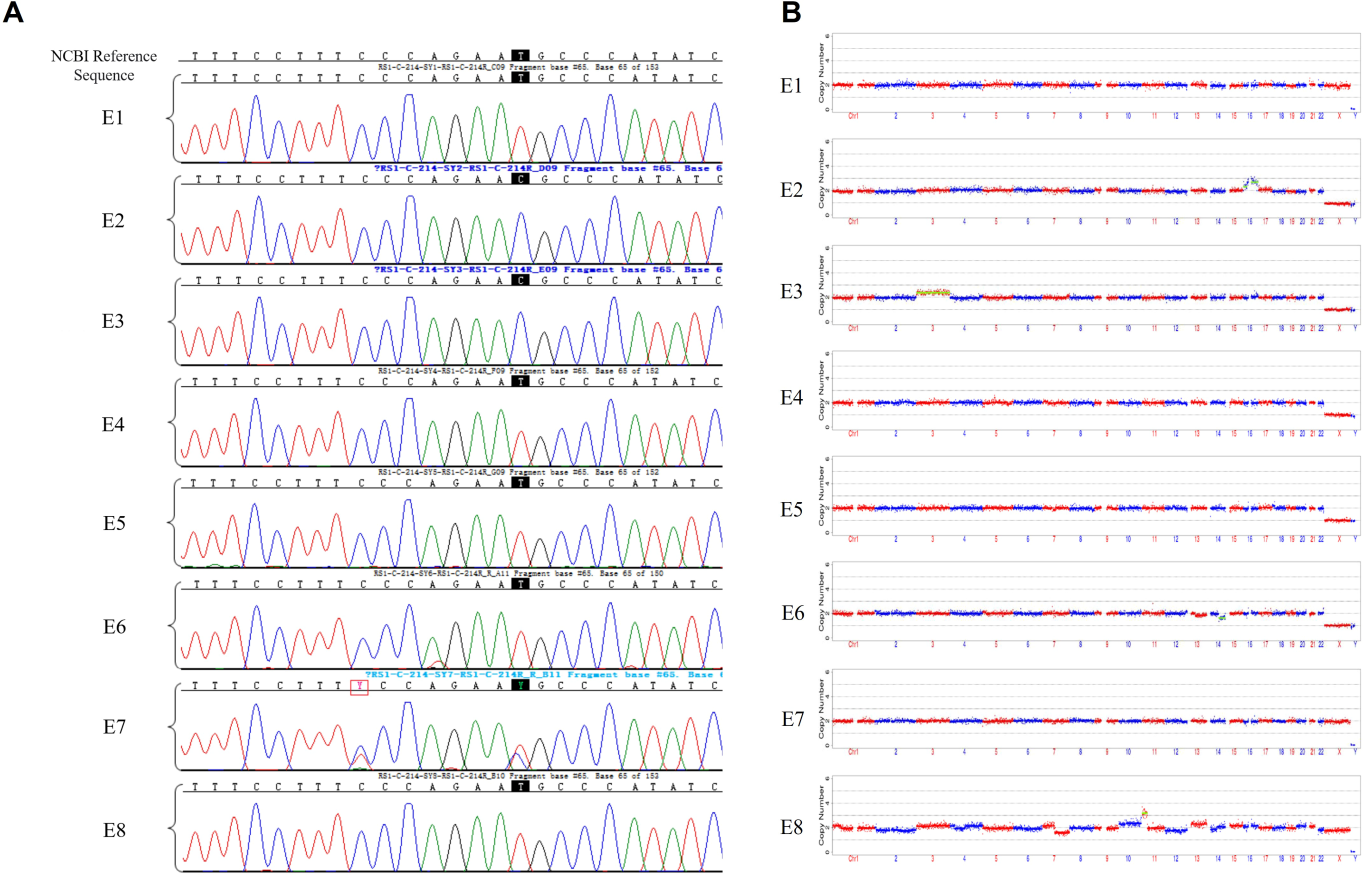
Sanger sequencing and CNV analysis of WGA products from embryonic trophoblast biopsy cells. **(A)** Sanger sequencing chromatograms of eight embryos. Mutation sites are shown as black boxes. The E1, E4, E5, E6, and E8 embryos did not inherit the pathogenic *RS1* mutation (p.Cys63Arg), while E2, E3, and E7 have the mutation; **(B)** CNVs in eight embryos, at a low NGS sequencing depth. NGS analysis of embryos E1, E4, E5, and E7 reveals no large CNVs or copy number abnormalities; however, embryos E2, E3, E6, and E8 show copy number abnormalities.

After ovulation stimulation, 11 oocytes were retrieved under ultrasonographic guidance. These metaphase II (MII) oocytes underwent ICSI fertilization, and all exhibited normal fertilization. Eight blastocysts were obtained on day six after ICSI and were graded according to the Gardner grading system. On day 6 after fertilization, we performed trophectoderm biopsy on all blastocysts graded > 3BB and collected 4–6 TE cells from each blastocyst. Embryo test results obtained using the MARSALA strategy. *RS1* in embryos E2, E3, and E7 contained the c.187T>C mutation, whereas no mutations were detected in E1, E4, E5, or E6 (**Figure 2A**). No CNVs larger than 4 Mb or aneuploidy were identified in embryos E1, E4, E5, or E7. However, E3 and E6 were mosaic embryos, whereas sequencing identified aneuploidy in embryos E2 and E8 (**Figure 2B**). To assess the reliability of our WGA and genotyping, we calculated the allelic dropout (ADO) rate. By analyzing the heterozygous SNP loci in the parental samples and their transmission to the embryos, the average ADO rate for this PGT-M cycle was determined to be 4.35%, which is within the acceptable range (<10%) for clinical PGT-M.

### 3.4 SNP haplotype analysis results

**Figure 3:**
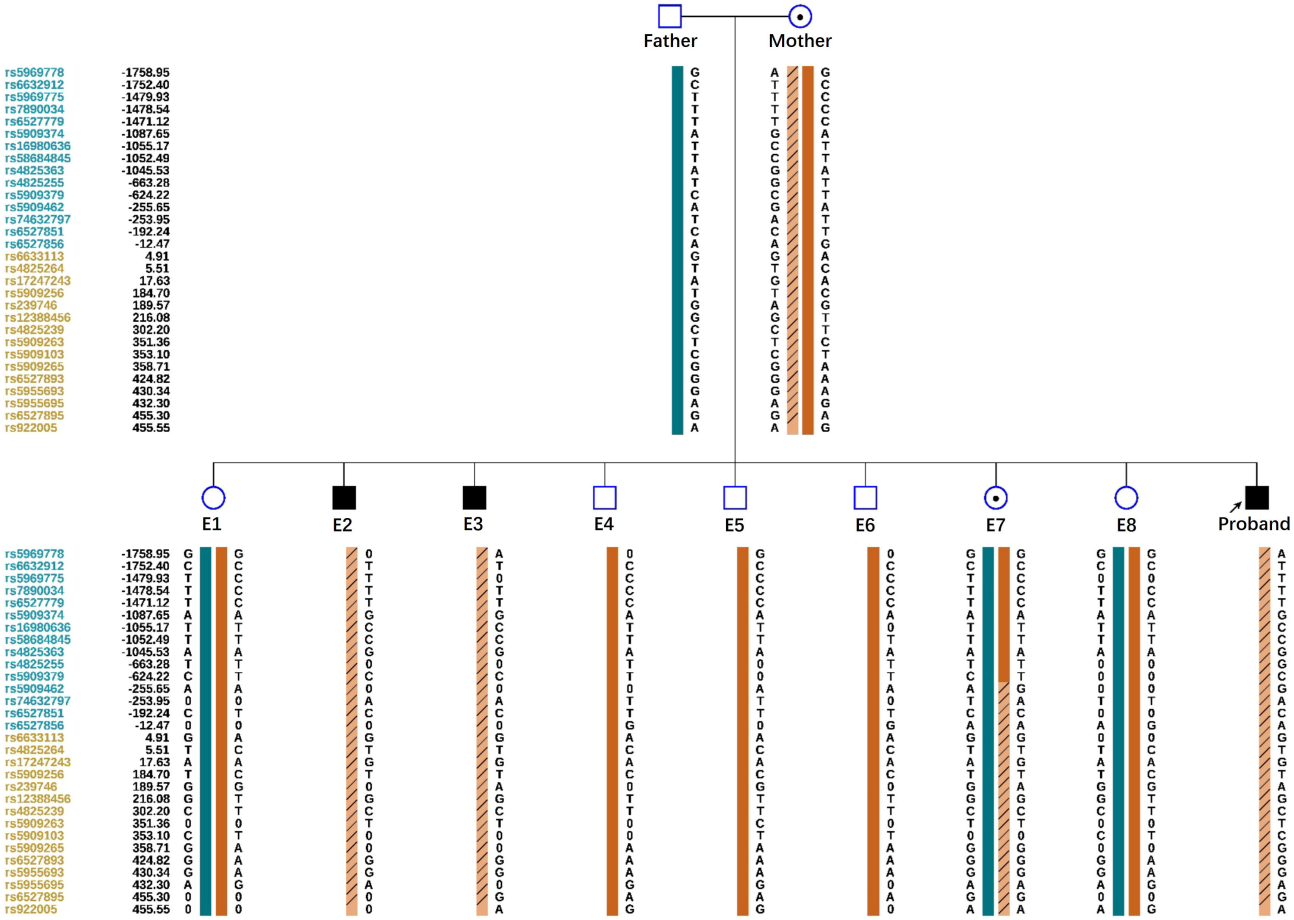
Schematic representation of the SNP-based haplotypes of family members and embryos. The SNP sites are indicated on the left, and blue and orange represent the SNP sites upstream and downstream, respectively, of the chromosomal position where the gene is located. Light orange with slashes represents high-risk haplotypes, dark blue bars represent normal or low-risk haplotypes in the father, and dark orange bars represent normal or low-risk haplotypes in the mother. The results show that the E1, E4, E5, E6 and E8 embryos are normal. E2, E3 and E7 embryos exhibits the maternal haplotype.

Linkage analysis was performed using SNP markers located within 2 Mb of *RS1* mutation. To establish haplotypes, 60 informative polymorphic SNPs were selected, and the analysis focused on 15 SNPs within 1 Mb upstream and downstream of the mutation site. The results of the family SNP haplotype analysis demonstrated the lack of maternal mutations within embryos E1, E4, E5, E6, and E8 (**Figure 3**). In addition, the positive results from pathogenic mutation detection and linkage analysis verified the absence of the *RS1* c.187T>C mutation in the aforementioned embryos.

### 3.5 Embryo transfer and prenatal genetic diagnosis

The optimal order for embryo transfer was determined via collaborative consultation between a reproductive physician, an embryologist, and a medical geneticist at the Reproductive Center of Zhongshan Bo’ai Hospital. An all-inclusive assessment of the embryo developmental stage and quality and results from Sanger sequencing, CNV analysis, and SNP haplotype analysis guided the unanimous decision to transfer embryo E1, which eventually resulted in a successful pregnancy (**Table 1**). In the 20th week of gestation, the pregnant woman underwent ultrasound-guided amniocentesis to obtain amniotic fluid cells for culture. Cultured cells were utilized for target fragment amplification, Chromosomal Microarray (CMA), and Sanger sequencing, which demonstrated results consistent with the PGT and the absence of any abnormalities. Eventually, a female infant, devoid of the mutation, was delivered. Postnatal ophthalmic screening performed at 3 months showed clear refractive media and normal fundus structure, consistent with the genetic diagnosis of a healthy female non-carrier.

**Table 1:** Summary of detection results.

Embryo number	Biopsy time	Gardner grade	Copy number variations	SNP haplotype	Sanger sequencing
E1	D6	6AA	46, XX	normal female	normal
E2	D6	6AA	46, XY, + 16p(p13.3→p12.2,~22Mb,×3,mos,~32%), +16p(p12.2→p11.2,~9Mb,×3), +16q(×3)	abnormal detection	c.187T>C
E3	D6	5AA	46, XY, + 3(×3,mos,~38%)	abnormal detection	c.187T>C
E4	D6	4AA	46, XY	normal male	normal
E5	D6	4AA	46, XY	normal female	normal
E6	D6	4AA	46, XY, -14q(q23.3→q32.33,~40Mb,×1,mos,~35%)	normal male	normal
E7	D6	4AB	46, XX	abnormal detection	c.187T>C
E8	D6	4AB	46, XX, + 11p(p15.5→p13,~31Mb,×3)	normal female	normal

## 4 Discussion

In this study, we report the case of a family with XLRS caused by a missense mutation in *RS1* on the X chromosome. Genetic analysis revealed a single-nucleotide missense mutation, c.187T>C (p.Cys63Arg; NM_000330.4), in exon 4 of *RS1*. This mutation involves a thymine (T) to cytosine (C) substitution at nucleotide 187, resulting in a change of cysteine (Cys) to arginine (Arg) at amino acid position 63 of the encoded protein. The highly conserved discoidin domain encoded by exon 4 is the primary functional component of the *RS1* protein and is crucial for maintaining the retinal structure and ensuring synaptic connections between its layers. Within the discoidin domain, the N-terminal Cys^63^ and C-terminal Cys^219^ can form intramolecular disulfide bonds, which is critical in protein folding (14). Previous studies have shown that substituting Cys^63^ or Cys^219^ with other amino acids causes a major portion of the expressed *RS1* protein to be retained in the endoplasmic reticulum as misfolded disulfide-bonded protein aggregates (14). The persistent accumulation of mutated *RS1* protein, both intracellularly and extracellularly, fails to adhere to the retinal layers, which may eventually lead to cystic changes and schisis formation in the retina (15). The c.187T>C mutation in *RS1* identified in this study was classified in the ClinVar database (https://www.ncbi.nlm.nih.gov/clinvar/) as a variant of uncertain significance/likely pathogenic (Variation ID: 1517929). In the present case, the *RS1* mutation in the proband met the ACMG/AMP criteria for PM2_supporting, PP3_strong, and PP4 rules, categorizing it as a “ Likely pathogenic”.

In this case, the mother is a carrier of a heterozygous *RS1* mutation, and her son carries the *RS1* mutation as a hemizygous carrier because of the location on Xq22. While symptomatic female carriers with a typical XLRS phenotype are exceedingly rare, they are often associated with homozygous *RS1* mutations or skewed X-chromosome inactivation (16). Recent large-scale case studies have highlighted extensive phenotypic variability in XLRS due to *RS1* mutations (6, 17), with no clearly established genotype-phenotype correlation. Even within the same family, patients with identical mutations may exhibit diverse phenotypes and natural histories (18), and asymmetrical phenotypes may manifest in the eyes of the same individual (6). The broad clinical spectrum resulting from the phenotypic variability poses significant challenges in the diagnosis and treatment of XLRS. Therefore, *RS1* mutations should be considered for young patients with unexplained ocular diseases. Direct sequencing of the *RS1* gene is crucial for the early diagnosis, genetic counseling, and clinical management of patients with suspected XLRS (14). Furthermore, promoting “carrier screening” among phenotypically normal populations is essential to identify more pathogenic gene carriers and prevent intergenerational transmission of disease-causing genes.

XLRS still lacks an effective treatment. The fundamental solution remains the prevention of *RS1* mutation intergenerational transmission. However, preventing *RS1* transmission via prenatal diagnostic techniques (amniocentesis), which involves termination of pregnancy, may cause psychological and physical harm to the pregnant woman. In PGT-M, genetic analysis can be performed using cells from a TE biopsy of the blastocyst, and unaffected embryos are selected for implantation into the mother’s uterus. This approach spares couples from having to decide on pregnancy termination or raising affected children, reducing the trial-and-error cost and shortening the time to parenthood.

In our practice, we used MARSALA-based PGT-M for the first time to block the intergenerational transmission of mutant *RS1* in a family with XLRS. Based on an expert consensus from Europe and China (19), the amplification efficiency of WGA in PGT should exceed 90%, and the ADO rate should be less than 10%. The core technology of MARSALA is MALBAC, which is not merely replication but also the selective amplification of the original genomic DNA by protecting the amplification products (20). Incomplete genome coverage is noticeable in events such as ADO, whereas MALBAC ensures excellent genome coverage and single-nucleotide variant (SNV) calling (20, 21). A study comparing five commercially available WGA kits determined that MALBAC has the lowest ADO rate (22). The products extended using MALBAC demonstrate good results for CNV analysis and SNP detection. Therefore, we utilized MALBAC for the genetic amplification of TE cells, marking the first application of MALBAC in XLRS. However, during PGT-M, the use of polymorphic markers from the proband and their parents, along with linkage analysis of the mutant gene to establish haplotypes, can further mitigate the impact of ADO (23–26). In the SNP linkage analysis, at least two polymorphic loci are selected that provide genetic information within 1 Mb upstream and downstream of the pathogenic mutation site, avoiding SNP loci with high homology, adjacent sequences, or high GC content in polynucleotide sequences. If the ADO rate is high, additional upstream and downstream-linked polymorphic loci can be analyzed. High-quality embryos are selected for morphological assessment using PGT-M. Through CNV, SNP haplotype, and Sanger sequencing analyses, we confirmed that embryos E1, E4, E5, and E7 did not carry the pathogenic RS1 p.(Cys63Arg) variant or chromosome abnormalities. Based on the PGT-M results and clinical genetic counseling, embryo E1 (6AA) was chosen for implantation, which resulted in a successful pregnancy. Even with PGT-M, chromosomal rearrangements, mosaicism, and ADO cannot be completely excluded. Therefore, mid-pregnancy amniocentesis is essential to reduce the risk of PGT-M misdiagnosis. In this case, genetic testing of amniotic fluid cells confirmed the PGT-M results, which led to the birth of a healthy female infant.

To our knowledge, this is one of the first reports of successful application of PGT-M to block transmission of the X-linked *RS1* gene. Similar PGT-M strategies have been successfully employed for other X-linked disorders, such as X-linked retinitis pigmentosa and blue cone monochromacy (27, 28), as well as for autosomal dominant/recessive retinal diseases like congenital cataract and primary congenital glaucoma (28). While the core principles of PGT-M are consistent, our case study emphasizes the successful application of a specific PGT-M technology to interrupt the inheritance of the *RS1* gene mutation. Our use of the MARSALA platform is advantageous because it yields highly uniform, high-coverage single-cell DNA amplification, a critical capability that substantially mitigates ADO risk. Importantly, MARSALA offers a comprehensive diagnostic solution, simultaneously enabling the detection of single-gene mutations, chromosomal abnormalities (PGT-A), and linkage information (10, 29). This integrated, multiplex approach effectively bypasses the inefficiencies and procedural burden of conventional multi-step assays, thereby enhancing throughput and providing a holistic evaluation of embryo health for optimal selection prior to transfer (10). This methodology has been previously validated in PGT-M for various monogenic conditions, including spinal muscular atrophy (30), Marfan syndrome (24), Coffin-Lowry syndrome (31), inborn errors of metabolism (32) and autosomal recessive cutis laxa (33).

Despite the significant advantages demonstrated in this case, the limitations of the MARSALA technique warrant consideration. First, technical and biological challenges remain. The reliance on linkage analysis necessitates accurate haplotype construction, which typically depends on the availability of genomic DNA from a proband or other informative family members. In cases involving *de novo* mutations or where family members are unavailable, establishing the linkage phase can be challenging, though strategies such as sperm sequencing or polar body analysis may serve as alternatives. Furthermore, the massive data output from high-throughput sequencing requires sophisticated bioinformatic pipelines and specialized personnel for data interpretation, which may limit the technique’s accessibility in basic clinical laboratories. In addition to technical constraints, cost is another critical factor. The implementation of NGS platforms and MALBAC amplification generally entails higher equipment and reagent costs than conventional PCR-based PGT-M methods, such as STR linkage analysis. However, conventional PGT-M often focuses solely on the monogenic disorder and requires a separate, additional assay if aneuploidy screening (PGT-A) is desired. MARSALA integrates mutation detection, linkage analysis, and PGT-A into a universal ‘one-step’ workflow. Therefore, while the single-run cost is higher, it may offer superior cost-effectiveness for patients requiring comprehensive screening by eliminating the need for dual biopsies or multiple testing platforms, and by potentially improving clinical outcomes through the exclusion of aneuploid embryos. Finally, it is important to note that while PGT-M base on MARSALA improves the genetic selection of embryos, the overall success of the procedure relies on standard IVF outcomes, which decline with advancing maternal age.

In summary, using MARSALA-based PGT-M, we successfully identified and selected high-quality embryos that were unaffected by the *RS1* mutation, and culminating in the birth of a healthy female infant unaffected by the XLRS. Our case underscores the viability of MARSALA-based PGT-M in halting the intergenerational transmission of *RS1* mutations and offers valuable insights into preventing birth defects from similar monogenic genetic diseases.

## Data Availability

The raw data supporting the conclusions of this article will be made available by the authors, without undue reservation.
